# Supplementary material for: Venomix: a simple bioinformatic pipeline for identifying and characterizing toxin gene candidates from transcriptomic data
Source: PeerJ. 2018 Jul 31;6:e5361. doi: 10.7717/peerj.5361 (PMC6074769; doi:10.7717/peerj.5361)
Supplement: Supplemental Information 6 [file peerj-06-5361-s006.gz › FinalOutput_1E-20/Phospholipase_A2_1/finaltree.pdf]

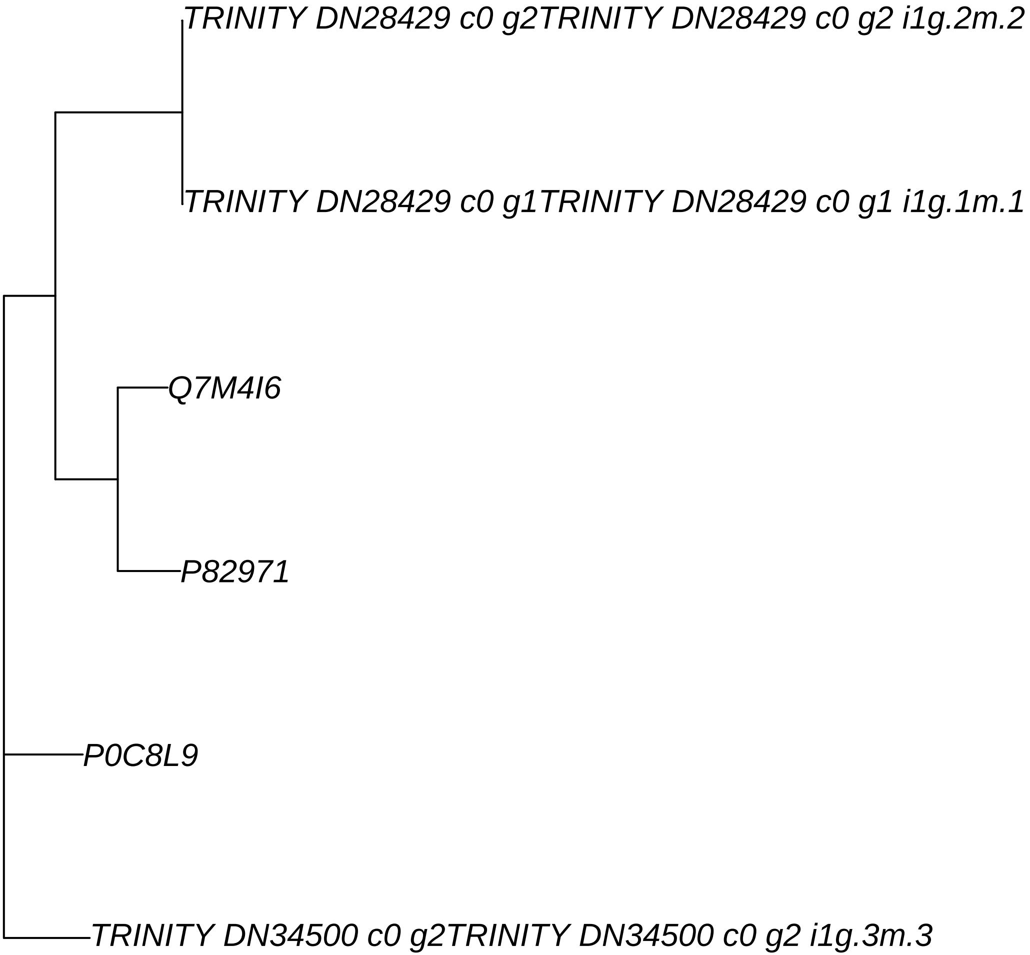
TRINITY DN28429 c0 g2TRINITY DN28429 c0 g2 i1g.2m.2
TRINITY DN28429 c0 g1TRINITY DN28429 c0 g1 i1g.1m.1
Q7M4I6
P82971
P0C8L9
TRINITY DN34500 c0 g2TRINITY DN34500 c0 g2 i1g.3m.3
